# A prognostic Bayesian network that makes personalized predictions of poor prognostic outcome post resection of pancreatic ductal adenocarcinoma

**DOI:** 10.1371/journal.pone.0222270

**Published:** 2019-09-09

**Authors:** Alison Bradley, Robert Van der Meer, Colin J. McKay

**Affiliations:** 1 Department of Management Science, Strathclyde Business School, University of Strathclyde, Glasgow, Scotland, United Kingdom; 2 West of Scotland Pancreatic Cancer Unit, Glasgow Royal Infirmary, Glasgow, Scotland, United Kingdom; University of South Alabama Mitchell Cancer Institute, UNITED STATES

## Abstract

**Background:**

The narrative surrounding the management of potentially resectable pancreatic cancer is complex. Surgical resection is the only potentially curative treatment. However resection rates are low, the risk of operative morbidity and mortality are high, and survival outcomes remain poor. The aim of this study was to create a prognostic Bayesian network that pre-operatively makes personalized predictions of post-resection survival time of 12months or less and also performs post-operative prognostic updating.

**Methods:**

A Bayesian network was created by synthesizing data from PubMed post-resection survival analysis studies through a two-stage weighting process. Input variables included: inflammatory markers, tumour factors, tumour markers, patient factors and, if applicable, response to neoadjuvant treatment for pre-operative predictions. Prognostic updating was performed by inclusion of post-operative input variables including: pathology results and adjuvant therapy.

**Results:**

77 studies (n = 31,214) were used to create the Bayesian network, which was validated against a prospectively maintained tertiary referral centre database (n = 387). For pre-operative predictions an Area Under the Curve (AUC) of 0.7 (*P* value: 0.001; 95% CI 0.589–0.801) was achieved accepting up to 4 missing data-points in the dataset. For prognostic updating an AUC 0.8 (*P* value: 0.000; 95% CI:0.710–0.870) was achieved when validated against a dataset with up to 6 missing pre-operative, and 0 missing post-operative data-points. This dropped to AUC: 0.7 (*P value*: 0.000; 95% CI:0.667–0.818) when the post-operative validation dataset had up to 2 missing data-points.

**Conclusion:**

This Bayesian network is currently unique in the way it utilizes PubMed and patient level data to translate the existing empirical evidence surrounding potentially resectable pancreatic cancer to make personalized prognostic predictions. We believe such a tool is vital in facilitating better shared decision-making in clinical practice and could be further developed to offer a vehicle for delivering personalized precision medicine in the future.

## Introduction

Pancreatic cancer is one of the most aggressive and challenging malignancies and is the fourth and fifth most common cause of cancer deaths in the USA and Europe respectively [1,2]. Overall 10-year survival of all cases diagnosed has remained at less than 1% despite advances in the fields of oncological therapies, surgical techniques and diagnostic technologies [3]. The reported percentage of cases amenable to surgical resection has been reported to be as low as 9.8% [3] and it is the narrative surrounding the management of potentially resectable cases of pancreatic cancer that is most complex, not least due to the ambiguities and controversies within the existing body of evidence.

The only potential cure for pancreatic cancer is surgical resection [3,4]. Adjuvant therapy has been proven to prolong survival with its role in the management of resected pancreatic cancer established through successive randomize controlled trials [4]. Therefore, surgery followed by adjuvant therapy has become the standard of care for resectable pancreatic cancer [4]. However such a narrative does not convey the full message contained within the empirical data.

Whilst it is true that surgical resection is the only potentially curative treatment, 5-year survival for resected cases of pancreatic cancer stands at between 7% and 25% [3]. Up to 50% of patients fail to receive adjuvant therapy due to post-operative complications, early disease reoccurrence and decline in function [5,6]. Consequently the potential benefits of such high-risk surgery, with its impact on quality-of-life, are often nullified.

The narrative becomes even more complex when considering the emerging role of neoadjuvant therapy as an alternative treatment pathway. Postulated benefits of this approach include: avoidance of futile surgery by identifying more aggressive tumours, eliminating micrometastatic disease to prevent early disease reoccurrence, increased R0 resection rates, and completion of multimodal treatment [7,8]. However, there is currently a lack of randomized controlled trials offering direct comparison between neoadjuvant and surgery-first approaches for resectable pancreatic cancer [9] and critics highlight the dangers of drawing optimistic conclusion regarding neoadjuvant therapy from a body of mainly small, underpowered studies [7,8]. Whilst the role of neoadjuvant therapy in the management of borderline resectable and locally advanced cases of pancreatic cancer has widely been accepted due to the potential benefits of conversion to resectability and achieving R0 resection [7,8], studies synthesizing the results of existing trials have reported only marginal benefits with neoadjuvant therapy [9–15]. Its role in the management of resectable pancreatic cancer remains controversial due to the potential risk of losing the window of resectability.

The question therefore arises as to how we can better communicate and transmit complex and data rich narratives to patients about their prognosis following a diagnosis of potentially resectable pancreatic cancer to facilitate better shared decision-making. Personalized predictive modeling, whereby patients are provided with forecasts of outcomes across competing treatment strategies, has gained precedence within contemporary medicine [16,17]. Its impact on the management of pancreatic cancer, considering the low surgical volume, high operative morbidity and mortality, and poor survival outcomes, could be significant through more effective patient counseling, risk-stratification and improved treatment selection [18]. However existing prognostic models fall short of achieving this goal and are seldom applied in the clinical settings [18]. The majority are based on single centre data which potentiates bias and limits generalizability, and few have undergone external validation. This is partly due to the fact that acquisition of large databases of potentially resectable cases is difficult as the majority of cases present with advanced disease [3]. Furthermore existing models predominately rely on post-operative data and are mainly based on traditional non-linear regression techniques which fail to encompass the dynamic nature of the care process whereby predicted outcomes evolve as events unfold, such as treatment complications, and time-dependent information emerges, such as post-operative pathological assessment of the resected tumour [19].

The aim of this study was to combine PubMed and patient level data to create and validate a prognostic Bayesian network that will make personalized predictions of poor prognostic outcome (defined as 12months or less survival time) post resection of pancreatic ductal adenocarcinoma (PDAC).

## Materials and methods

### Bayesian network

Based on probability theory, Bayesian networks (BN) model relationships between variables based on a graphical formalism of a joint or multivariate probability distribution over a set of variables. This is formalized as: BN = (*G*,*Pr)*. *G* is a graphical structure and *Pr* is the probability distribution [16,19,20–22]. Within the graphical structure of a BN, *G*, variables are modeled as nodes (V(G)) with causal relationships between parent and child nodes represented by directed arcs (A(G)) therefore *G* = V(G), A(G). Within a BN any number of nodes can be included therefore V(G) = {V_1,_ V_2_….V_n_} where *n*>1. Directed arcs, A(G), represent the probabilistic influence between parent (V_i_)and child (V_j_) nodes: V_i_ V_j_ [16,19,20–22].

The dependence and independence between nodes is defined by the joint probability distribution (*Pr*):

$$\mathrm{Pr}\left(xi\ldots .xn\right)=\prod i\;Pr(\frac{xi}{pai})$$

where *x*_*i*_ represents the value of variable x_i_ and *pa*_*i*_ represents a set of values for the parents of x_i_ which gives the conditional probability distribution[16,19,20–22]. Furthermore each variable within the network is independent of non-descendent nodes:

Pr(x4|x1,x2,x3)=Pr(x4|x2,x3)

whereby x_2_ and x_3_ are the parents of x_4_ which is independent of x_1_ therefore each node has a conditional probability table representing the probability of each value contained within that node given the condition of all its parent nodes [20–22]. Through Bayes theorem the prior distribution and observed data are combined to update knowledge in the form of the posterior distribution [20]. Missing data is handled through probabilistic inference with predictions made based on global averages of the patient population [19,22]. In this way BN allow the modeling of the dynamic relationships between variables contained within the complex healthcare process, with predictions evolving and accuracy improving as more information becomes available [17,19].

### Evidence synthesis

PubMed database was searched following the PRISMA guidelines [23] (S1 Fig) with the entire database included from 1^st^ January 2000 up to and including 3^rd^ December, 2018 using the full list of search terms provided in supplementary material (S1 Table). The inclusion criteria were full-text multivariable survival analysis studies of patients aged 18years or over that had undergone resection of PDAC whether treated in neoadjuvant or upfront surgery pathways. Included studies had to report the results of survival analysis of patients who had undergone resection of PDAC where the aim of the study was to identify variables associated with a post resection survival time of 12months or less. Studies that included other subtypes of pancreatic cancer and observational and cohort studies that reported only survival outcomes without multivariate analysis of variables associated with the survival outcome in question were excluded. Reference lists and citations of all included papers were also manually screened to identify any additional articles. This was repeated until no new articles were identified. All manuscripts were assessed using a combination GRADE guidelines [24], and Zhu et al. [25] checklist of items for evaluating the quality of reporting of survival-analysis. Studies of poor quality and with a high risk of bias were excluded.

The first author performed search design and data extraction. The second author performed independent data extraction and quality assurance. Data was extracted manually from studies and included: study year, number of included patients, risk-of-bias information, all variables that were included in the multivariate analysis, and for each variable whether it was found to have a statistically significant association with poor prognosis post resection of PDAC as defined by a P value <0.005. Inter-reviewer discussion resolved any discrepancies. This yielded 77 papers, giving a pool of n = 31,214 patients from which the model was built (S2 Table).

Adapting methods from Zhao and Weng [26], extracted data underwent a two stage weighting process. The original weight for each variable (*w*^o^_*i*_) represents a summary of existing evidence, including conflicting findings [26], and was calculated as *w*^o^_*i*_ = P_*i*_/N_i_ where N_i_ represents the total number of times in the body of evidence that the variable was included in multivariate analysis and P_*i*_ represents the number of times where the variable was found to be statistically significant in its association with a poor post resection prognosis. A process of normalization, adapted from Zhao and Weng [26], was then undertaken to place this ratio in the context of the entire PubMed body of evidence related to each variable and poor post resection prognostic outcome. Normalized weights, *w*_*i*_, were defined as:

$$wi=w{}^{\circ}i(\frac{\max\left(pw{}^{\circ}1,pw{}^{\circ}2,\ldots pw{}^{\circ}n\right)}{max(ps1,ps2,\ldots psn)})$$

The sum of the study populations reporting the variable, (*pw*^*0*^), is defined as max(*pw*^o^_*1*,_ *pw*^o^_*2*,_ …. *pw*^o^_*n*_). The sum of the study populations of all included studies is defined as max(*ps*_1_, *ps*_2_,…*ps*_*n*_). Both weights are therefore defined on a scale of 0 to 1, with the normalized weighting used to rank each variable in order of significance (Table 1).

**Table 1:** Weighted variables from synthesized PubMed studies (n = 31214).

Variable/ Node	Node Status	Definition	Rank based on normalized weighting
Lymph Node Positive	Yes No		1
Lymph node ratio	<0.3 >0.3	Ratio of the number of positive lymph nodes to the total number of lymph nodes removed	2
Tumour Grade	G1/G2 G3/G4	As per American Joint Committee on Cancer definition [19]: Well/moderate differentiation, low/intermediate grade Poorly differentiated, high grade	3
Tumour Size	< 2cm >2cm		4
R0 Resection	No Yes	No microscopic evidence of any residual tumour	5
Adjuvant Therapy	No Yes		6
T stage	T1 T2 T3 T4		7
Pre treatment Ca 19–9	<50 50–999 >1000	<50 U/mL 50–999 U/mL >1000 U/ mL	8
AJCC (American Joint Committee on Cancer) Stage	0 1 2 3 4	As per AJCC definition	9
Vascular Involvement	Yes No		10
Perineural Involvement (PNI)	Yes No		11
Age	< 70 >70	Under 70 years Equal to or over 70 years	12
mGPS (modified Glasgow Prognostic Score)	0 1 2	0 = CRP</ = 10mg/L and albumin >/ = 35 g/L 1 = CRP > 10mg/L 2 = CRP> 10mg/L and albumin <35 g/L	13
CEA>5	<5 >5	<5 ng/mL >5 ng/mL	14
Performance Status	Good Moderate Poor	As defined by American Society of Anaestheologits (ASA) classification ASA 1–2 ASA 2–3 ASA >3	15
Tumour Location	HOP Body/Tail	Head of Pancreas (HOP) Location other than HOP	16
Post treatment Ca19-9	<120 >120	<120 U/mL >120 U/mL	17
Prei operative Blood Transfusion	Yes No		18
Albumin	Normal Low	= /> 35 g/l < 35 g/l	19
Neutrophil Lymphocyte Ratio	<5 >5		20
Jaundice	No Yes	Bilirubin < 40μmol/l Bilirubin >40μmol/l	21
Diabetes	No Yes		22
Smoking	Non-smoker Smoker		23
Response to Neoadjuvant Treatment	Stable Progression/ Unresectable	Radiological response or stable disease that is still resectable Radiological evidence of progression/ unresectable disease	24
BMI	Normal Low	Body Mass Index (BMI) above 18 BMI equal or under 18	25

### Bayesian network structure

**Fig 1:**
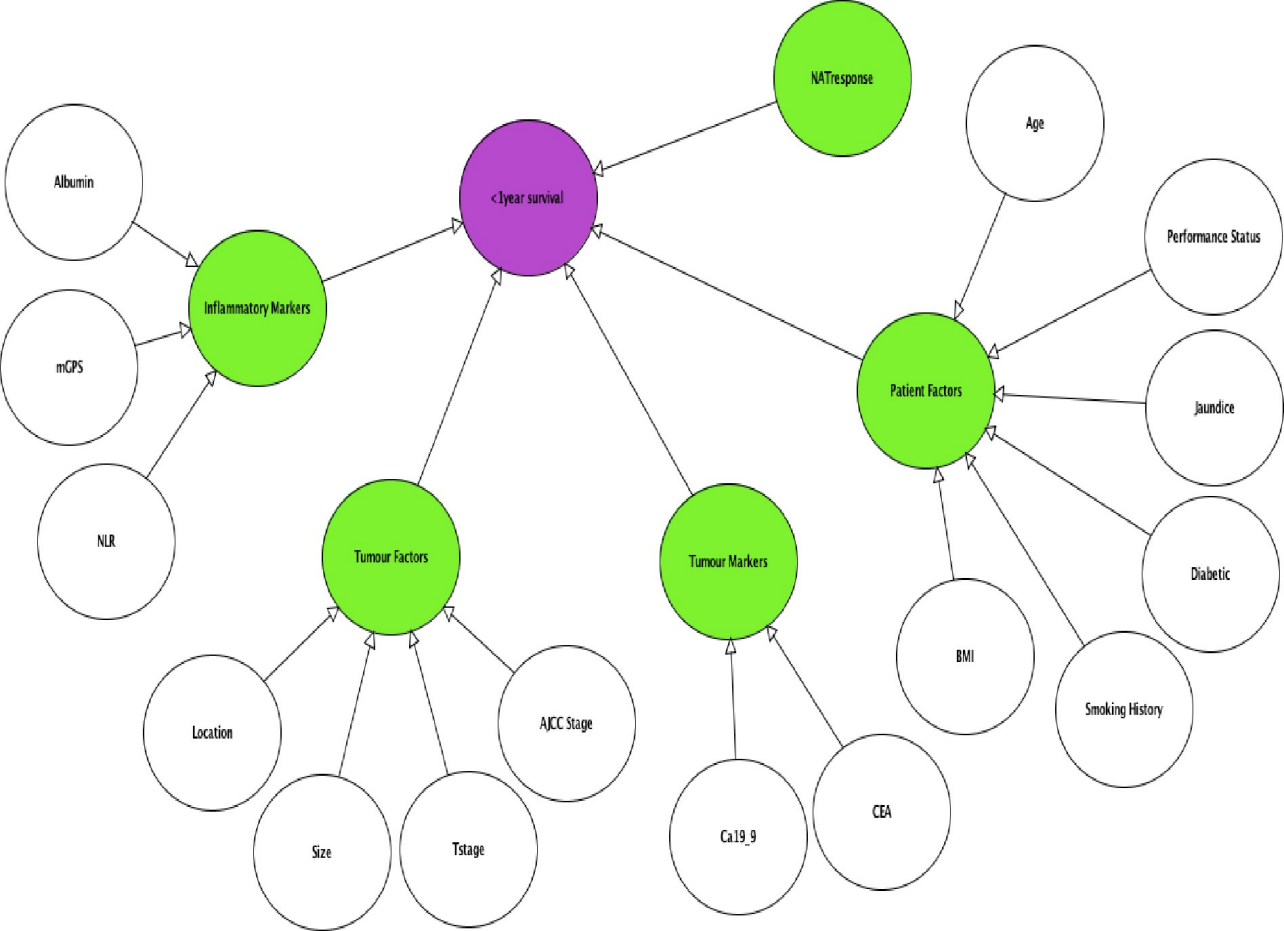
Bayesian network to predict poor post resection prognosis. Parent nodes in white, child nodes in green and output node in purple.

**Fig 2:**
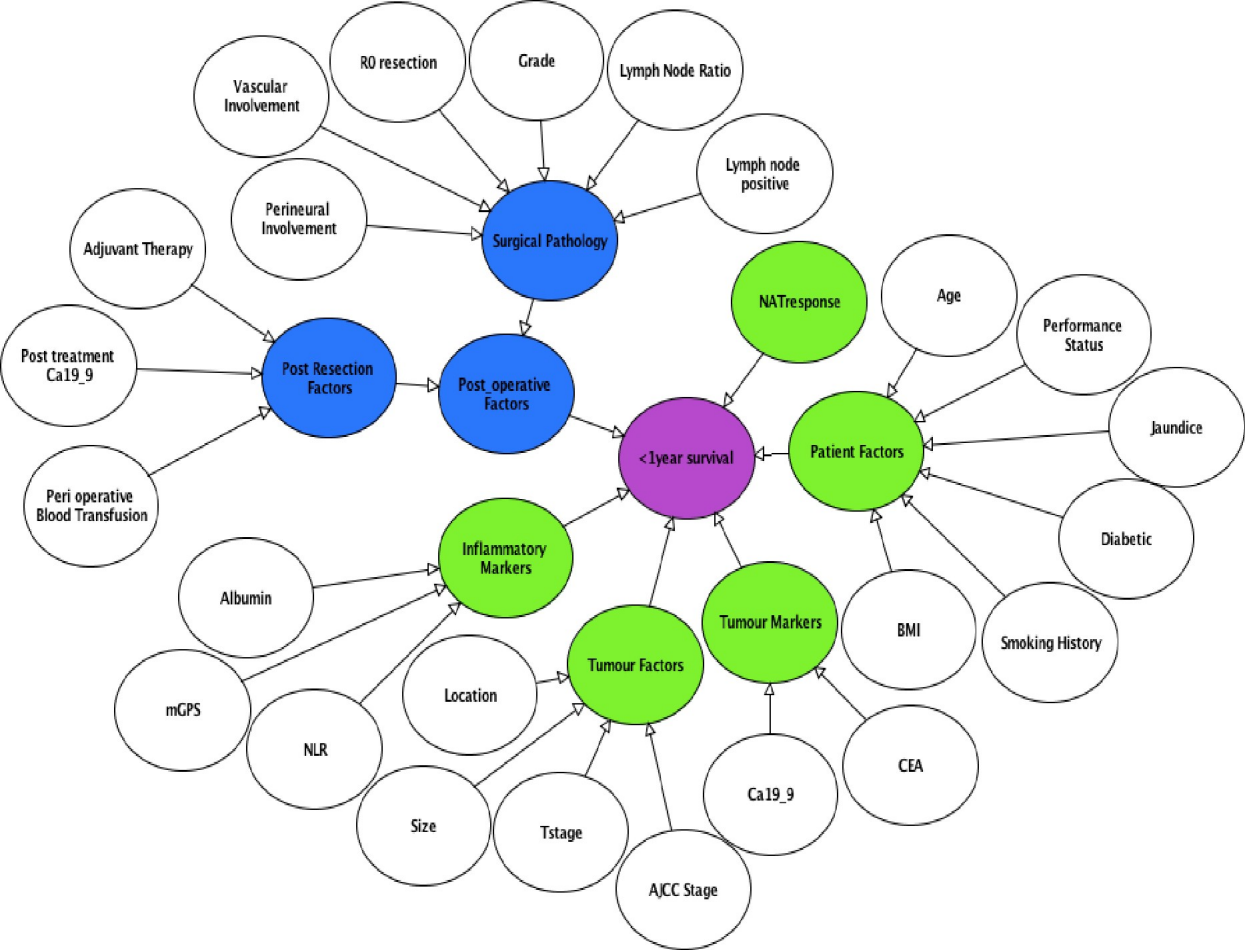
BBN to predict poor prognosis post resection. Pre-operative and post-operative parent nodes in white, pre-operative child nodes in green, post-operative child nodes in blue and output node in purple.

The top 25 ranking variables (Table 1) were used to structure the BN created using AgenaRisk version 7.0 software [27]. Variables known pre-operatively were used to construct the pre-operative version of the model (Fig 1) with post-operative variables added to perform prognostic updating (Fig 2). Each variable was treated as a ranked parent node and were linked to their respective child nodes through causative arcs.

To calculate the node probability table for each child node, the normalized weighting of each parent node was used as the weighted mean of the truncated Normal (TNormal) distribution. The final output node was a Boolean node whereby a 50% or greater probability of ‘yes’ for 1year survival or less was accepted as predicting poor prognosis post resection. It was calculated from the weighted mean of the corresponding parent nodes. The TNormal statistical distribution was used as it has been proven to generate accurate node probability tables for BN involving ranked nodes with ranked parent nodes [20].

The definitions and categorization of input data for each node within the BN are detailed in Table 1. These definitions and categorizations were determined by how this data was presented in the published studies and they, as well as the overall model structure, were approved by an expert panel of pancreatic surgeons.

### Model validation

The performance of the model was assessed using the area under the curve (AUC) of the received operated curve (ROC) using SPSS Statistics version 24 software. It was validated against a 20year, prospectively maintained patient database from a tertiary referral centre. Individual patient data was entered into the BN and the personalized pre and post-operative predictions of poor prognosis were recorded and assessed against that individual’s actual survival time therefore deeming predictions to be true or false. All patients who had survival data recorded, had died, or if still alive had a survival time beyond 12 months, and who, for the post-operative BN had post-operative data available, were included. Patients who were found to have non-resectable disease at operation, or who were treated in a neoadjuvant pathway and were found to have non-resectable disease at re-staging, were included to reduce the risk of bias when validating the pre-operative BN as in the clinical setting the intention would have been to perform resection. This gave a pool of 387 and 251 patients against which the predictive performance of the pre and post-operative models were validated respectively.

## Results

### Pre-operative predictions

**Fig 3:**
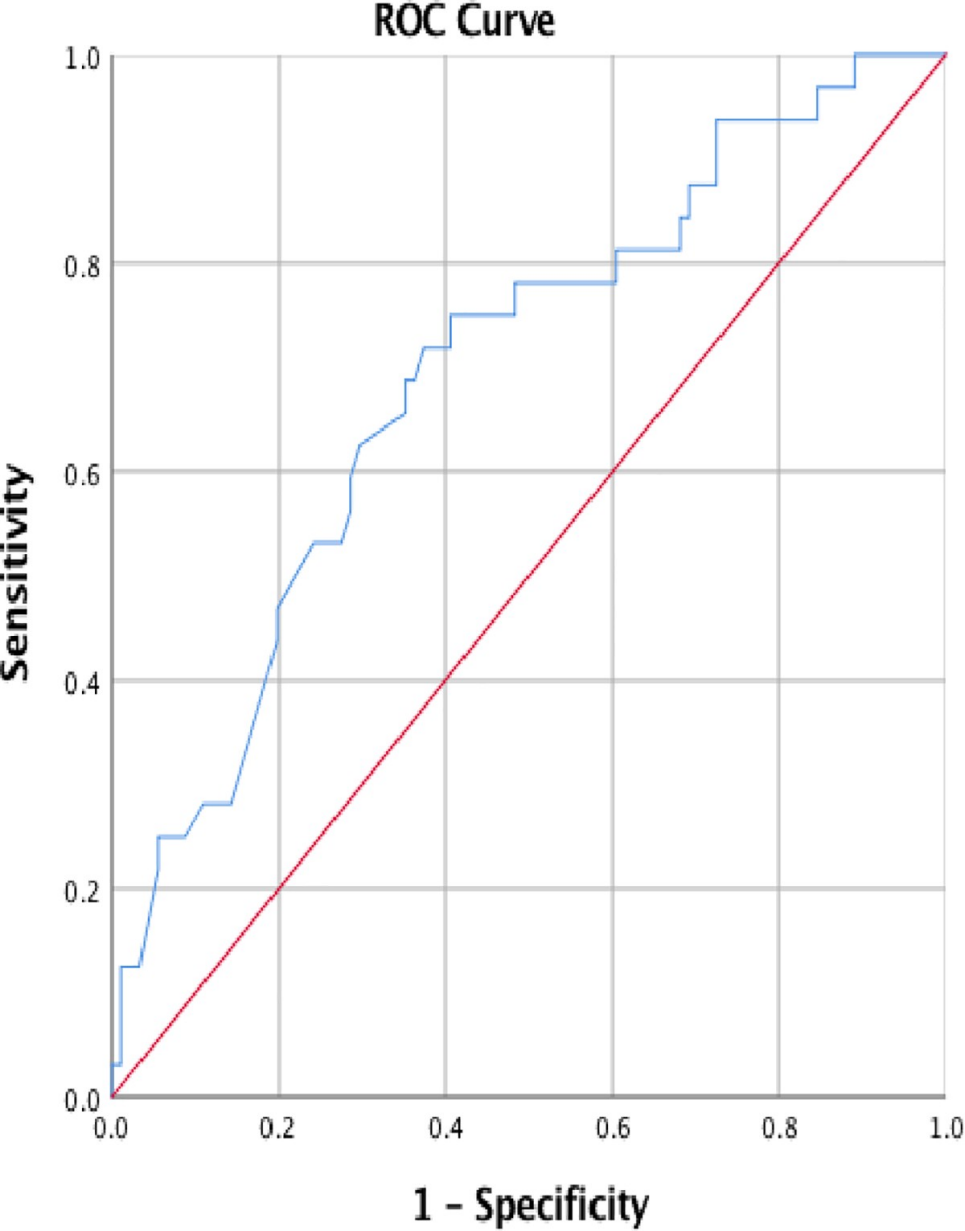
Receiver operated curve (ROC) for pre-operative predictions where all data was available apart from tumour markers.

The database against which the BN was validated did not contain data on tumour markers Ca19-9 and CEA, which were the third and seventh ranked pre-operative nodes respectively (Table 1). Despite this missing data the model achieved an AUC of 0.7 (*P* value 0.001; 95% CI 0.589–0.801) where data on all other nodes were available (Fig 3). A statistically significant AUC of 0.7 was maintained when an additional one and two data points were missing (Table 2). At the point where an additional three data points were missing the AUC remained above 0.6 but lost statistical significance (Table 2).

**Table 2:** Results of model performance in handling missing data.

Validation Dataset	AUC
2 data points missing (n = 123)	0. 7 (*P* value 0.001; 95% CI 0.589–0.801) Std. Error: 0.54
3 data point missing (n = 139)	0. 7 (*P* value 0.001; 95% CI 0.578–0.786) Std. Error: 0.53
4 data points missing (n = 144)	0.7 (*P* value 0.001; 95% CI 0.591–0.791) Std. Error: 0.51
5 data points missing (n = 176)	0. 6 (*P* value 0.009; 95% CI 0.537–0.711) Std. Error: 0.44
6 data points missing (n = 189)	0.6 (*P* value 0.024; 95% CI 0.518–0.690) Std. Error: 0.44
6+ data points missing (n = 387)	0.6 (*P* value 0.559; 95% CI 0.502–0.617) Std. Error: 0.29

### Prognostic updating

**Fig 4:**
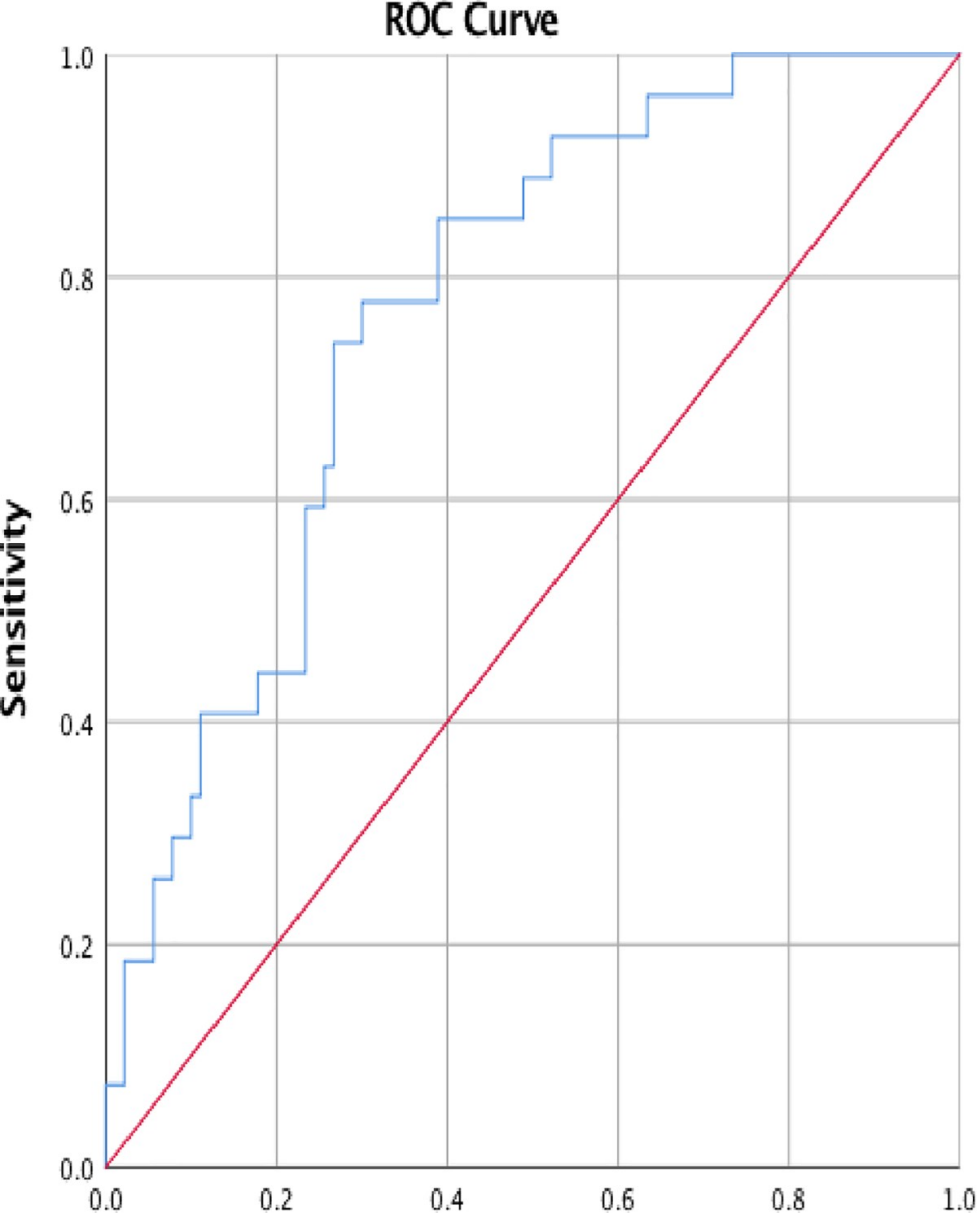
Receiver operated curve (ROC) for post-operative predictions where all data was available apart from tumour markers.

In addition to the absence of data on Ca19-9 and CEA in the pre-operative validation dataset, data on post-treatment Ca19-9 levels, the 17^th^ highest ranked variable (Table 1) was also missing from the validation dataset. Despite this the post-operative model maintained an AUC of 0.8 (*P value*: 0.000; 95% CI: 0.678–0.862) when all other data was available (Fig 4). An AUC of 0.8 was maintained until greater than 6 pre-operative data points, and up to and including 2 post-operative data points, were missing which resulted in an AUC of 0.7 (*P value*: 0.000; 95% CI:0.667–0.818) (Table 3). An AUC of 0.7 was then maintained even when the validation data set could contain over 6 missing pre-operative data points and up to and including 4 missing post-operative data points (*P value*: 0.000; 95% CI: 0.660–0.788).

**Table 3:** Summary of performance for prognostic updating.

	1 Missing Post-operative Data points	1–2 Missing Post-operative Data Point	1–3 Missing Post-operative Data Points	1–4 Missing Post-operative Data Points
**2 Missing Pre-operative Data Points**	AUC 0.8; Standard Error:0.47; *P value*: 0.000; 95% CI: 0.678–0.862 (n = 117)	AUC: 0.8; Standard Error:0.51; *P value*: 0.000; 95% CI: 0.651–0.850 (n = 120)		
**2–3 Missing Pre-operative Data Point**	AUC: 0.8; Standard Error:0.045; *P value*: 0.000; 95% CI: 0.685–0.862 (n = 138)	AUC: 0.8; Standard Error:0.045; *P value*: 0.000; 95% CI: 0.685–0.862 (n = 139)		
**2–4 Missing Pre-operative Data Points**	AUC: 0.8; Standard Error: 0.042; *P value*: 0.000; 95% CI: 0.708–0.872 (n = 135)	AUC: 0.8; Standard Error: 0.045; *P value*: 0.000; 95% CI: 0.681–0.858 (n = 140)		
**2–5** **Missing Pre-operative Data Points**	AUC: 0.8; Standard Error: 0.041; *P value*: 0.000; 95% CI: 0.708–0.869 (n = 137)	AUC: 0.8; Standard Error: 0.043; *P value*: 0.000; 95% CI: 0.681–0.849 (n = 146)		
**2-6 Missing Pre-operative Data Points**	AUC: 0.8; Standard Error:0.041; *P value*: 0.000; 95% CI: 0.707–0.869 (n = 138)	AUC: 0.8; Standard Error:0.043; *P value*: 0.000; 95% CI: 0.665–0.832 (n = 155)	AUC: 0.8; Standard Error:0.042; *P value*: 0.000; 95% CI: 0.672–0.835 (n = 157)	
**>6 Missing Pre-operative Data Points**	AUC: 0.8; Standard Error:0.041; *P value*: 0.000; 95% CI: 0.710–0.870 (n = 139)	AUC: 0.7; Standard Error:0.039; *P value*: 0.000; 95% CI: 0.667–0.818 (n = 195)	AUC: 0.7; Standard Error: 0.037; *P value*: 0.000; 95% CI: 0.667–0.814 (n = 205)	AUC: 0.7; Standard Error:0.033; *P value*: 0.000; 95% CI: 0.660–0.788 (n = 251)

## Discussion

By utilizing existing PubMed data in a unique way within a BN, prognostic predictions of 12months or less survival time post resection of PDAC were made at the pre-operative stage of the patient journey with an AUC of 0.7 even when up to 4 data points were missing. The BN also demonstrated its ability to perform prognostic updating at the post-operative stage of the patient journey with an AUC of 0.8, which was maintained even when greater than 6 pre-operative, and 1 post-operative, data points were missing. Above this threshold for missing data an AUC of 0.7 was achieved.

The performance of the BN compares favorably to existing predictive model development studies aiming to predict poor post pancreatic cancer resection prognosis. Existing models based on multivariate cox proportional hazard regression techniques report an AUC of between 0.7 and 0.887 [28–31]. However many are based on single institution databases [28,29,31] and failed to undergo external validation [28,29] One study, based on single institution data, used artificial neural network technique to predict 7month mortality post-resection and reported an AUC of 0.6576 but did not perform external validation [32]. One study used Bayesian modeling techniques and National Registry data to predict 6month, 1,3 and 5year survival and achieved a c-statistic of 0.65 [33].

### Strengths and limitations

This model is unique on several levels. Firstly the creation of a BN allowed the novel utilization of knowledge from existing PubMed studies in a clinically more meaningful way for individual patients and their clinicians. This also means that the model, based on the wider collective body of existing evidence, overcomes the limitations of many existing models that lack generalizability as they are largely based on single institutional database analysis with the potential for inherent bias that this creates. This also allows the BN to make predictions even when data is missing through probabilistic inference with predictions made based on global averages of the patient population [19,22]. Secondly this model goes beyond the few existing nomograms and prognostic models by providing personalized predictions based on pre-operative information therefore being of more value in patient counseling and decision-making throughout the patient journey. Thirdly this model is unique in its ability to make personalized predictions of outcome across the competing treatment strategies of upfront surgery and neoadjuvant approach.

One limitation of this model is that it is based on published survival analysis studies, which are predominately single centre studies that also carry a risk of bias. Whilst the two-stage process of weighting variables was designed to minimize the potential impact of such bias on the BN, there is also the potential that new and emerging studies will alter the weightings of nodes within the BN, particularly as the role of neoadjuvant therapy becomes more established with its impact on survival time consequently becoming more widely analysed. The model would also benefit from being validated against another institution’s database, particularly considering that data on tumour markers was absent from the validation dataset used in this study. These nodes therefore relied on probabilistic inference to make predictions. It is a strength of the model that an AUC of 0.8 was achieved under these circumstances. Considering that tumour markers were the 8^th^, 14^th^ and 17^th^ ranked variables, it is possible that the performance of the BN could be even stronger had data on tumour markers been available. To address this and further refine the BN, the next phase will be to incorporate patient level data from large international patient databases into the existing model so that the accuracy of predictions can be further improved by combining the prior distribution and observed data to update the posterior distribution through Bayes theorem [20].

### Study impact

This study marks a potentially significant step towards to achieving the delivery of personalized cancer care. In the clinical setting the BN presented here has the potential to have an immediate impact on improving patient counseling and facilitating better shared decision-making by providing a mechanism to communicate and transmit the complex and data rich empirical narrative surrounding a diagnosis of potentially resectable pancreatic cancer to patients on a personalized level. This includes being better able to explain the impact of “what if” scenarios on anticipated prognosis such as not achieving R0 resection, or not receiving adjuvant therapy even if R0 resection is achieved.

The second area of impact of this study is in directing future research. As patient databases globally develop in complexity and mature, so too should predictive modeling become more sophisticated at integrating multiple complex databases to make individualized patient predictions and support clinical decision-making [34–37]. This coincides with the growing interest in precision medicine where it is anticipated that our understanding of disease at genomic level will lead to gene-targeted therapies [35,36]. However, patients are more than their genomes. By developing a predictive model that can integrate clinical, pathological, and biochemical data to make meaningful personalized predictions of outcomes, this BN paves the way for future versions of this BN to incorporate emerging genomic data hence creating a vehicle for delivering truly personalized precision medicine and accelerating the clinical application of our ever expanding knowledge-base [37].

## Supporting information

S1 Fig PRISMA flowchart. (TIF)

S1 Table Search terms. (DOCX)

S2 Table Summary of included studies. (XLSX)

S3 Table Tripod checklist. (DOCX)
