# Supplementary figures and images for: A prognostic Bayesian network that makes personalized predictions of poor prognostic outcome post resection of pancreatic ductal adenocarcinoma
Source: PLoS One. 2019 Sep 9;14(9):e0222270. doi: 10.1371/journal.pone.0222270 (PMC6733484; doi:10.1371/journal.pone.0222270)

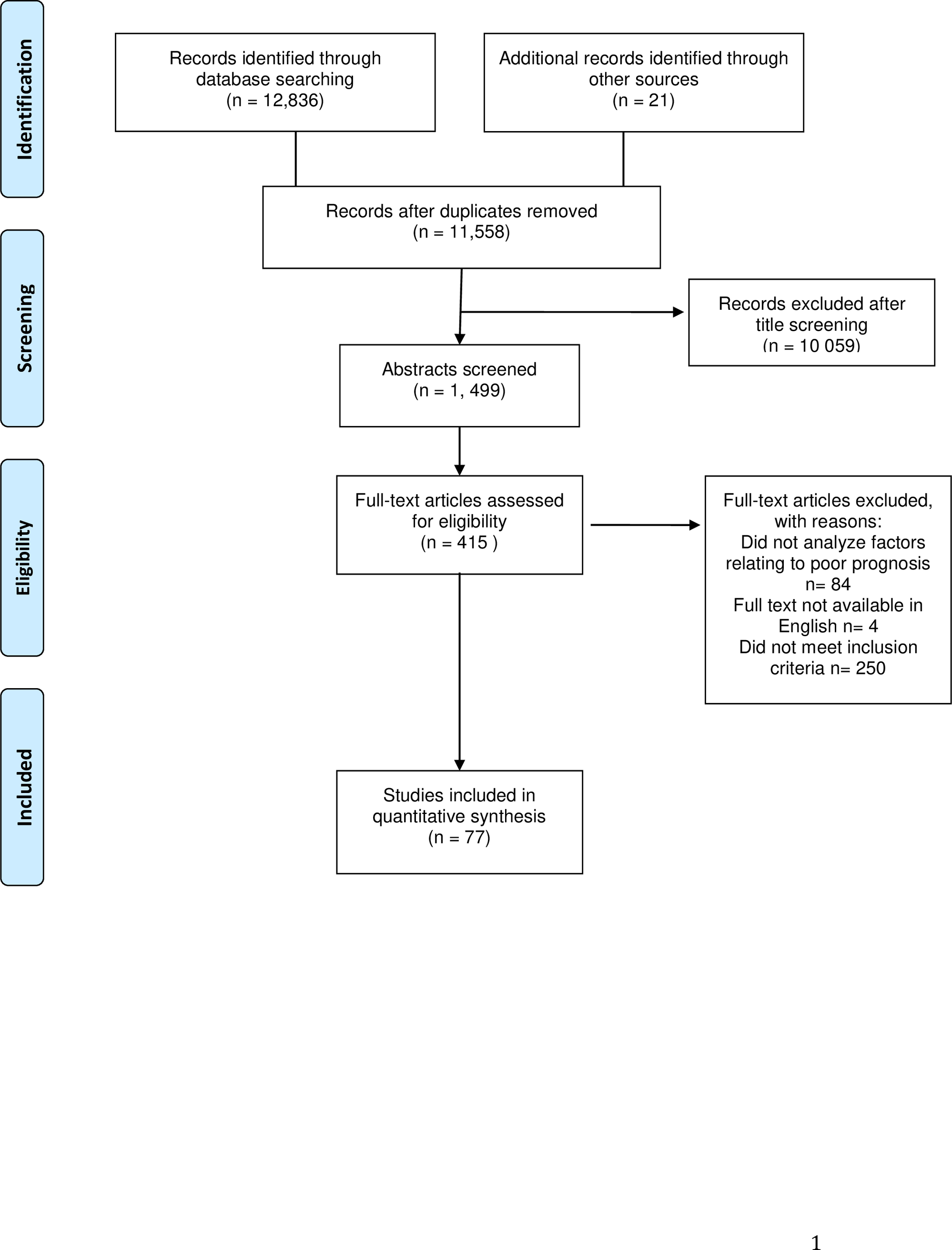

Supplement: S1 Fig — (TIF) [file pone.0222270.s001.tif]
