# Supplementary material for: A prognostic Bayesian network that makes personalized predictions of poor prognostic outcome post resection of pancreatic ductal adenocarcinoma
Source: PLoS One. 2019 Sep 9;14(9):e0222270. doi: 10.1371/journal.pone.0222270 (PMC6733484; doi:10.1371/journal.pone.0222270)
Supplement: S1 Table — (DOCX) [file pone.0222270.s002.docx]

| Pancreatic cancer survival analysis {Including Related Terms} |
| --- |
| - pancreatic - pancrea - pancreas - cancer - cancers - neoplasm malignant - neoplasm cancer - malignant tumoral disease - malignant tumor - malignant neoplasms - malignant neoplasm - malignancies - malignancy - malignant tumour - malignant neoplastic disease - tumor malignant - tumour malignant - unclassified tumor malignant - unclassified tumour malignant - cancer morphology - malignant tumor morphology - malignant tumour morphology - primary malignant neoplasm - survival analysis - survival analyses - analysis survival - analyses survival |
| Pancreatic cancer prognostic prediction {Including Related Terms} |
| - prognostic - prediction - predictions - pancreatic cancer - cancer pancreas - cancer pancreatic - cancers pancreas - cancers pancreatic - malignant neoplasm pancreas - pancreas cancer - pancreas cancers - pancreatic cancers - malignant tumor of pancreas - malignant tumour of pancreas - pancreatic carcinoma - pancreas carcinoma - exocrine pancreas carcinoma |
| pancreatic cancer post resection survival {Including Related Terms} |
| - resection - excision - excisions - surgical resection - surgical removal - ectomy - removal - extirpation - res - abscission - incision and removal - excision procedure - re section - re sections - resections - survival - continuance of life - survivorship issues - survivorship - survival aspects - survivals - pancreatic cancer - cancer pancreas - cancer pancreatic - cancers pancreas - cancers pancreatic - malignant neoplasm pancreas - pancreas cancer - pancreas cancers - pancreatic cancers - malignant tumor of pancreas - malignant tumour of pancreas - pancreatic carcinoma - pancreas carcinoma - exocrine pancreas carcinoma |
| pancreatic cancer post resection prognosis {Including Related Terms} |
| post  after values  dorsal  dorsals  dossal  dossals  dossel  back  posterior  back of  retro  posts  trading post  trading posts  resection  excision  excisions  surgical resection  surgical removal  ectomy  removal  extirpation  res  abscission  incision and removal  excision procedure  re section  re sections  resections  prognosis  courses  prognoses  forecast of outcome  determination of prognosis  outcome prediction  pancreatic cancer  cancer pancreas  cancer pancreatic  cancers pancreas  cancers pancreatic  malignant neoplasm pancreas  pancreas cancer  pancreas cancers  pancreatic cancers  malignant tumor of pancreas  malignant tumour of pancreas  pancreatic carcinoma  pancreas carcinoma  exocrine pancreas carcinoma |
